# Supplementary material for: Telomere repeats induce domains of H3K27 methylation in Neurospora
Source: eLife. 2018 Jan 3;7:e31216. doi: 10.7554/eLife.31216 (PMC5752202; doi:10.7554/eLife.31216)
Supplement: Supplementary file 1. — The numbers and their corresponding genotypes are indicated for all strains used in this study. [file elife-31216-supp1.docx]

**Supplementary File 1. List of strains**

| Strain | Genotype |
| --- | --- |
| N51 (FGSC 2225) | *mat A;* Mauriceville |
| N625 | *mat a; his-3* |
| N2834 | *mat A; his-3*; Δ*mus-52::hph^+^* |
| N2931 | *mat a;* Δ*mus-52::bar^+^* |
| N3752 (FGSC 2489) | *mat A;* Oak Ridge |
| N4730 | *mat A;* Δ*set-7::bar^+^* |
| N4933 | *mat a*; Δ47.4 kb::*hph^+^* |
| N5100 (FGSC 1614) | *mat a;* In(IL->IR)AR16 |
| N5101 (FGSC 2100) | *mat A;* T(IV->VI)ALS159 |
| N5102 (FGSC 3670) | *mat A;* T(VI->III)OY329 |
| N5547 | *mat a;* Δ*mus-52::bar^+^;* Δ47.4 kb::*hph^+^* |
| N5683 | *mat A*; *his-3^+^::1;* Δ47.4 kb::*hph^+^* |
| N5684 | *mat A*; *his-3^+^::2;* Δ47.4 kb::*hph^+^* |
| N5685 | *mat A*; *his-3^+^::3;* Δ47.4 kb::*hph^+^* |
| N5686 | *mat A*; *his-3^+^::4;* Δ47.4 kb::*hph^+^* |
| N5687 | *mat A*; *his-3^+^::5;* Δ47.4 kb::*hph^+^* |
| N5688 | *mat A*; *his-3^+^::6;* Δ47.4 kb::*hph^+^* |
| N5689 | *mat A*; *his-3^+^::7* Δ47.4 kb::*hph^+^* |
| N5690 | *mat A*; *his-3^+^::8;* Δ47.4 kb::*hph^++^* |
| N5695 | *mat a*; Δ*csr-1::1;* Δ47.4 kb::*hph^+^* |
| N5696 | *mat a*; Δ*csr-1::2;* Δ47.4 kb::*hph^+^* |
| N5697 | *mat a*; Δ*csr-1::3;* Δ47.4 kb::*hph^+^* |
| N5698 | *mat A*; Δ*csr-1::4;* Δ47.4 kb::*hph^+^* |
| N5699 | *mat A*; Δ*csr-1::5;* Δ47.4 kb::*hph^+^* |
| N5700 | *mat a*; Δ*csr-1::6;* Δ47.4 kb::*hph^+^* |
| N5701 | *mat a*; Δ*csr-1::7;* Δ47.4 kb::*hph^+^* |
| N5702 | *mat A*; Δ*csr-1::8;* Δ47.4 kb::*hph^+^* |
| N5739 | *mat A*; *his-3;* Δ47.4 kb::*hph^+^* |
| N5857 (FGSC 1483) | *mat A;* T(II->V)NM149 |
| N5858 (FGSC 3668) | *mat A;* T(II->IV)OY337, *al-2* |
| N5859 (FGSC 7294) | *mat A;* T(IVR->VL)UK2-32 |
| N5862 (FGSC 4641) | *mat A;* T(VIL->IR)OY350 |
| N5863 (FGSC 3635) | *mat A;* T(VI->III)OY320 |
| N5866 (FGSC 6869) | *mat A; inl*; T(VR )UK3-41 |
| N6089 | *mat a;* Dp(VI->III)OY329 |
| N6093 (FGSC 3671) | *mat a;* T(VI->III)OY329 |
| N6228 | *mat A;* Δ*tert::nat-1^+^* |
| N6381 | *mat a;* Δ*mus-52::bar^+^;* Δ*csr-1::*(TTAGGG)_8_ |
| N6383 | *mat a;* Δ*mus-52::bar^+^;* Δ*csr-1::*(TTAGGG)_17_ |
| N6984 | *mat a;* Δ*mus-52::bar^+^;* Δ*csr-1::*(TTAGGG)_8_ |
| N6985 | *mat a;* Δ*mus-52::bar^+^;* Δ*csr-1::*(TTAGGG)_8_ |
| N6986 | *mat a;* Δ*mus-52::bar^+^;* Δ*csr-1::*(TTAGGG)_17_ |
| N6987 | *mat a;* Δ*mus-52::bar^+^;* Δ*csr-1::*(TTAGGG)_17_ |
| N7515 | *mat A;* Δ*mus-52::hph^+^; his-3^+^::*(TTAGGG)_23_ (heterokaryon) |
| N7516 | *mat A;* Δ*mus-52::hph^+^; his-3^+^::*(TTAGGG)_22_ (heterokaryon) |
| N7517 | *mat A;* Δ*mus-52::hph^+^; his-3^+^::*(TTAGGG)_21_ (heterokaryon) |
