# Supplementary material for: Telomere repeats induce domains of H3K27 methylation in Neurospora
Source: eLife. 2018 Jan 3;7:e31216. doi: 10.7554/eLife.31216 (PMC5752202; doi:10.7554/eLife.31216)
Supplement: Supplementary file 2. — All primers included in this study are identified by primer number, a brief description of how they were used and their corresponding sequence. [file elife-31216-supp2.docx]

**Supplementary File 2. List of primers**

| Primer | Description | Sequence |
| --- | --- | --- |
| 3325 | Construction of *his- 3+::1* (N5683) | CGGGATCCCCGGATCGAACGGCGGATGG (BamHI) |
| 3326 | Construction of *his- 3+::1* (N5683) | GCTCTAGATGAGCTCCTTCCCCCACGGT (XbaI) |
| 3327 | Construction of *his- 3+::2* (N5684) | CGGGATCCCGTGGGTTCCAGTGCGTCCC (BamHI) |
| 3328 | Construction of *his- 3+::2* (N5684) | GCTCTAGATGGCAAGCGCCGACATGTGA (XbaI) |
| 3329 | Construction of *his- 3+::3* (N5685) | GGGCCCAGGGAACAGGCGCAGTGCAG (ApaI) |
| 3330 | Construction of *his- 3+::3* (N5685) | GACTAGTTGGCGCGCTTGACCAGCAAA (SpeI) |
| 3331 | Construction of *his- 3+::4* (N5686) | GACTAGTTCCCGGAACGGCCACTCCAT (SpeI) |
| 3332 | Construction of *his- 3+::4* (N5686) | GCTCTAGAGTGCTGCCAAGGCCCGACAT (XbaI) |
| 3297 | Construction of *his-3+::5* (N5687) | GGAATTCCAAAGGATCGCGCCCGGAGG (EcoRI) |
| 3333 | Construction of *his-3+::5* (N5687) | CGGGATCCACGACGGGAAGACGAGGGGT (BamHI) |
| 3334 | Construction of *his- 3+::6* (N5688) | GGGCCCGGGAGTGTGGCGCTGTGGAC (ApaI) |
| 3335 | Construction of *his- 3+::6* (N5688) | GACTAGTAGCAGCATCTCGGGTCCGGT (SpeI) |
| 3301 | Construction of *his- 3+::7* (N5689) | GGAATTCGGCATGCCCAGGGATTGGGG (EcoRI) |
| 3336 | Construction of *his- 3+::7* (N5689) | GCTCTAGAGACGGAACGGTGACAGGCGG (XbaI) |
| 3303 | Construction of *his- 3+::8* (N5690) | GGAATTCTGGTTCGCGGACGAGGGCTA (EcoRI) |
| 3337 | Construction of *his- 3+::8* (N5690) | CGGGATCCGGCTATTGCTGCCAGCCGGT (BamHI) |
| 4654 | 3’ *csr-1* RP | AACACCTCCGTCGCCATAAACTCC |
| 4655 | 5’ *csr-1* FP | GGCCCCTGGTTTACTGAGGGC |
| 4721 | 5’ *csr-1* RP | TGCAGCCATTGACGACATTGC |
| 4722 | 3’ *csr-1* FP | TGGATTTCCTGCGCTGCACAC |
| 4723 | Construction of Δ*csr-1::1* (N5695) | GCAATGTCGTCAATGGCTGCACCGGATCGAACGGCGGATGG |
| 4724 | Construction of Δ*csr-1::1* (N5695) | GTGTGCAGCGCAGGAAATCCATGAGCTCCTTCCCCCACGGT |
| 4725 | Construction of Δ*csr-1::2* (N5696) | GCAATGTCGTCAATGGCTGCACGTGGGTTCCAGTGCGTCC |
| 4726 | Construction of Δ*csr-1::2* (N5696) | GTGTGCAGCGCAGGAAATCCATGGCAAGCGCCGACATGTGA |
| 4727 | Construction of Δ*csr-1::3* (N5697) | GCAATGTCGTCAATGGCTGCAAGGGAACAGGCGCAGTGCAG |
| 4728 | Construction of Δ*csr-1::3* (N5697) | GTGTGCAGCGCAGGAAATCCATGGCGCGCTTGACCAGCAAA |
| 4729 | Construction of Δ*csr-1::4* (N5698) | GCAATGTCGTCAATGGCTGCATCCCGGAACGGCCACTCCAT |
| 4730 | Construction of Δ*csr-1::4* (N5698) | GTGTGCAGCGCAGGAAATCCAGTGCTGCCAAGGCCCGACAT |
| 4731 | Construction of Δ*csr-1::5* (N5699) | GCAATGTCGTCAATGGCTGCACAAAGGATCGCGCCCGGAGG |
| 4732 | Construction of Δ*csr-1::5* (N5699) | GTGTGCAGCGCAGGAAATCCAACGACGGGAAGACGAGGGGT |
| 4733 | Construction of Δ*csr-1::6* (N5700) | GCAATGTCGTCAATGGCTGCAGGGAGTGTGGCGCTGTGGAC |
| 4734 | Construction of Δ*csr-1::6* (N5700) | GTGTGCAGCGCAGGAAATCCAAGCAGCATCTCGGGTCCGGT |
| 4735 | Construction of Δ*csr-1::7* (N5701) | GCAATGTCGTCAATGGCTGCAGGCATGCCCAGGGATTGGGG |
| 4736 | Construction of Δ*csr-1::7* (N5701) | GTGTGCAGCGCAGGAAATCCAGACGGAACGGTGACAGGCGG |
| 4737 | Construction of Δ*csr-1::8* (N5702) | GCAATGTCGTCAATGGCTGCATGGTTCGCGGACGAGGGCTA |
| 4738 | Construction of Δ*csr-1::8* (N5702) | GTGTGCAGCGCAGGAAATCCAGGCTATTGCTGCCAGCCGGT |
| 4784 | qChIP *his-3* & *csr-1* targeting P1Fwd | AATGCAAGGTCCCGAACACT |
| 4785 | qChIP *his-3* & *csr-1* targeting P1Rev | TGGCTGTCGCAATTACCAGT |
| 4786 | qChIP *his-3* & *csr-1* targeting P2Fwd | GACCAAGCATGCGTTAGCTG |
| 4787 | qChIP *his-3* & *csr-1* targeting P2Rev | ACCCAAGGTGGGTGTGTTTT |
| 4788 | qChIP *his-3* & *csr-1* targeting P3Fwd | CCGTTTGAGCTGGTCTTCCT |
| 4789 | qChIP *his-3* & *csr-1* targeting P3Rev | TGACGGATGCTCTTTGTCCC |
| 4790 | qChIP *his-3* & *csr-1* targeting P4Fwd | CAACCAGCTTGACGGCTTTC |
| 4791 | qChIP *his-3* & *csr-1* targeting P4Rev | TACCGTAGGTGCCCTGTGTA |
| 3317 | qChIP *his-3* & *csr-1* targeting P5Fwd and deletion analysis | CCCCTTCCTGCCGTGGGAGA |
| 3562 | qChIP *his-3* & *csr-1* targeting P5Rev and deletion analysis | TCAGCAGGCATAGTCAAGACTGGT |
| 4782 | qChIP *his-3* & *csr-1* targeting P6Fwd | CCCGCTTCAGCAACCAAGTT |
| 4783 | qChIP *his-3* & *csr-1* targeting P6Rev | AACTTTAGCCCGCGTTACGG |
| 3319 | qChIP *his-3* & *csr-1* targeting P7Fwd and deletion analysis | TGGGTCGATGGAGTACCTTCCCC |
| 3563 | qChIP *his-3* & *csr-1* targeting P7Rev and deletion analysis | TGCACTATCCTTTTCAGGGGCTTGT |
| 3902 | qChIP *his-3* & *csr-1* targeting P8Fwd | GACCTACACGGCCCGGGGAA |
| 3903 | qChIP *his-3* & *csr-1* targeting P8Rev | ACCGACGAGACTTGACTGCCCA |
| 3986 | qChIP *his-3* & *csr-1* targeting P9Fwd | GTGGCGGCGTGAACGGTCAT |
| 3987 | qChIP *his-3* & *csr-1* targeting P9Rev | AGTCAAGCCTCGCGATCGTGA |
| 3321 | qChIP *his-3* & *csr-1* targeting P10Fwd | TGAACAGGTGACGGCGGGAGT |
| 3564 | qChIP *his-3* & *csr-1* targeting P10Rev | CGGGTCCGGAGTCCATCACCA |
| 3976 | qChIP *his-3* & *csr-1* targeting P11Fwd | AGTGGTCCAGAGTGGGATCGGT |
| 3977 | qChIP *his-3* & *csr-1* targeting P11Rev | ACCGCCAATATGGCATCGCCC |
| 3974 | qChIP *his-3* & *csr-1* targeting P12Fwd | TGACGGCGCGCAGATTGGAG |
| 3975 | qChIP *his-3* & *csr-1* targeting P12Rev | TCGCTTCCCTTCTCCCACCATCC |
| 3978 | qChIP *his-3* & *csr-1* targeting P13Fwd | TTGACGGCGCGCAGATTGGAG |
| 3979 | qChIP *his-3* & *csr-1* targeting P13Rev | CCACCATCCTTCCCTCTGCCACA |
| 3992 | qChIP *his-3* & *csr-1* targeting P14Fwd | CATCGCAGCTCAACCGCAGA |
| 3993 | qChIP *his-3* & *csr-1* targeting P14Rev | GCCAGCCGGTGTCAAGACAGA |
| 3904 | qChIP *his-3* & *csr-1* targeting P15Fwd | AACAAAGACGCTCTTCTGGTGGCC |
| 3905 | qChIP *his-3* & *csr-1* targeting P15Rev | ACTACCAAACTGCCGACGGCT |
| 3943 | Construction of Δ47.4 kb::*hph^+^*(N4933) | GTAACGCCAGGGTTTTCCCAGTCACGACGTGTGGCTTGCAGGCACGCAA |
| 3944 | Construction of Δ47.4 kb::*hph^+^*(N4933) | ACCGGGATCCACTTAACGTTACTGAAATCAGTCCGAGTGGGCCTGCCTC |
| 3945 | Construction of Δ47.4 kb::*hph^+^*(N4933) | gctccttcaatatcatcttctgtcgacggACCACCACCCAGCGTGGAAAG |
| 3946 | Construction of Δ47.4 kb::*hph^+^*(N4933) | GCGGATAACAATTTCACACAGGAAACAGCTTGCCGCCGGCTGAGAAACC |
| 4960 | ALS159 (N5101) qPCR | TTGGGATGATTTGGGACGGG |
| 4961 | ALS159 (N5101) qPCR | TCCCAAGCTGACAGTTCCAC |
| 4961 | ALS159 (N5101) qPCR | TGCATGCTCTCCCCCTTTTG |
| 4963 | ALS159 (N5101) qPCR | TCTGAGGGATGTGCCAAACC |
| 4974 | NM149 (N5857) qPCR | CGCCATTTCTACCCCGATGA |
| 4975 | NM149 (N5857) qPCR | TGCCAAGCCATCTTTTTGCC |
| 4976 | NM149 (N5857) qPCR | CTACGGGTTGCTGCCAAGTA |
| 4977 | NM149 (N5857) qPCR | CCTCAGAGAATCGGGGCATC |
| 4978 | NM149 (N5857) qPCR | GGGCTCAGTCACTTGCTACA |
| 4979 | NM149 (N5857) qPCR | GATATACCCGCACCAGCACA |
| 4984 | OY337 (N5858) qPCR | CTTCGCCTCTCACTCCGATG |
| 4985 | OY337 (N5858) qPCR | GGCAGCTAGCAATCGGTTTT |
| 4986 | OY337 (N5858) qPCR | GAGCCTGTCCAAGACGACAA |
| 4987 | OY337 (N5858) qPCR | CGGTGACGGTAGTGTGTAGG |
| 4990 | OY337 (N5858) qPCR | GTTACTGGCGGGAAATGGGA |
| 4991 | OY337 (N5858) qPCR | TTGGGACCAGGTTTGTCCAC |
| 4996 | OY350 (N5862) qPCR | GCCTTGGACCCTCGAATGAA |
| 4997 | OY350 (N5862) qPCR | TGGGAAAACGTGGGGGAAAA |
| 4998 | OY350 (N5862) qPCR | TGGGTGAGGTCTTTGGAGGA |
| 4999 | OY350 (N5862) qPCR | AAGAGTTCCTGAACGTCGCC |
| 5002 | OY350 (N5862) qPCR | AGGGTTGCTGGTAATCCGTG |
| 5003 | OY350 (N5862) qPCR | CAAGGCTTGGGGAAAGGGAA |
| 3317 | UK3-41 (N5866) qPCR | CCCCTTCCTGCCGTGGGAGA |
| 3562 | UK3-41 (N5866) qPCR | TCAGCAGGCATAGTCAAGACTGGT |
| 3954 | UK3-41 (N5866) qPCR | GTAGCTAGCGGGTGCTGCCG |
| 3955 | UK3-41 (N5866) qPCR | AGGCGCCAGGAAGAGTATAGCCC |
| 5012 | OY329 (N5102) qPCR | AAATCCACTCATCCTCGGCG |
| 5013 | OY329 (N5102) qPCR | CTCGGATCACCGTCAACAGG |
| 5020 | OY329 (N5102) qPCR | ATGGTAACGTGGACAGGTGC |
| 5021 | OY329 (N5102) qPCR | TTGAACGCCGTAGAGGGATG |
| 5028 | UK2-32 (N5859) qPCR | AGGAAGTACGCCTTGCAGTC |
| 5029 | UK2-32 (N5859) qPCR | CCTGTATAATGGCGGTCCCC |
| 5034 | UK2-32 (N5859) qPCR | TCGAACCATGTGAGCTGCTT |
| 5035 | UK2-32 (N5859) qPCR | GAGAACGCCGAATCGCTCTA |
| 5036 | AR16 (N5100) qPCR | GAGAACGCCGAATCGCTCTA |
| 5037 | AR16 (N5100) qPCR | GCCCCCTTTTTGTCGTTAGC |
| 5040 | AR16 (N5100) qPCR | CGTCAACGGTAGCTGGAAGA |
| 5041 | AR16 (N5100) qPCR | CCTCTTTGTGTCGAAGCCCA |
| 5042 | AR16 (N5100) qPCR | TGCCAAAGCACAACAAGCTG |
| 5043 | AR16 (N5100) qPCR | TTGATACCACGGGCTTCGAC |
| 5046 | AR16 (N5100) qPCR | GGATCATCGGTAGGTTGGGT |
| 5047 | AR16 (N5100) qPCR | TCAGATCCAGCTAGTTTCGCC |
| 5316 | Telomere repeat FP | ttagggttagggttaggg |
| 5317 | Telomere repeat RP | ccctaaccctaaccc |
| 5318 | Targeting tel repeats to *csr-1* FP1 | gcaatgtcgtcaatggctgcaattaaccctcactaaaggga |
| 5319 | Targeting tel repeats to *csr-1* RP1 | gtgtgcagcgcaggaaatccataatacgactcactataggg |
| 5320 | Targeting tel repeats to *csr-1* FP2 | gtgtgcagcgcaggaaatccaattaaccctcactaaaggga |
| 5321 | Targeting tel repeats to *csr-1* RP2 | gcaatgtcgtcaatggctgcataatacgactcactataggg |
| 5322 | Verify LG I circularization FP | AGAGGAGTCCGTAGGCGAAT |
| 5323 | Verify LG I circularization RP | TCGTTCGGTTGACAGCTTGA |
| 5324 | Verify LG II circularization FP | TGTTTCGGCGATGGGAAGAA |
| 5325 | Verify LG II circularization RP | ACTTCGAGTATGTAGCGGCG |
| 5326 | Verify LG III circularization FP | CGAGGCTCCATAATGCTCGT |
| 5327 | Verify LG III circularization RP | TATTATAGGGCGCGCGGAAG |
| 5328 | Verify LG IV circularization FP | GGCGCAAAAACCTTCCTACC |
| 5329 | Verify LG IV circularization RP | ACGACAGGGCCTAGGGTAAT |
| 5330 | Verify LG VI circularization FP | TAGGTTGAAGGCTATCGGCG |
| 5331 | Verify LG VI circularization RP | CCTTGGTTGCATTTGGTGGG |
| 5332 | Verify LG VII circularization FP | GCCTTCGGCTACCTTTCCTT |
| 5346 | Verify LG VII circularization RP | CTCCCTTTCAGCTCGTGTGT |
| 5353 | 3’ *csr-1* qPCR FP | cgccgttaatgcagttgtgat |
| 5354 | 3’ *csr-1* qPCR RP | ccccagcaactgcgtctatt |
| 5133 | NM149 (N5857) breakpoint 1 FP | TTGCGGCAAGTTTGAAGTCG |
| 5134 | NM149 (N5857) breakpoint 1 RP | TGAAGCGTAAGCTCGTGTGT |
| 5130 | NM149 (N5857) breakpoint 2 FP | GCTCAAAGTGGGGACTGACA |
| 5154 | NM149 (N5857) breakpoint 2 RP | ATCCTTCTCCGCTGTTTCGG |
| 5123 | OY329 (N5102) breakpoint 1 FP | GTTGTTGTGGTTTCCTCGCC |
| 5124 | OY329 (N5102) breakpoint 1 RP | ATATAGGCGTAGCGTTGCCC |
| 5125 | OY329 (N5102) breakpoint 2 FP | TGTTGCCTGGACTGCTAGTG |
| 5126 | OY329 (N5102) breakpoint 2 RP | AGCCTAAACCTCGGCTAGGA |
| 5127 | OY329 (N5102) breakpoint 3 FP | CCGGTATCACGAGCTTCTCC |
| 5128 | OY329 (N5102) breakpoint 3 RP | GGGCGGAAGTTGAGCTGTAT |
| 5155 | UK3-41 (N5866) breakpoint 1 FP | GAACGGGACGTTCAAGGCTA |
| 5157 | UK3-41 (N5866) breakpoint 1 RP | TGCTTGTCTCGTTTTGCAGC |
| 5158 | UK3-41 (N5866) breakpoint 2 FP | CGGGAGAGGGGGATAGTTGA |
| 5473 | UK3-41 (N5866) breakpoint 2 RP | CGCACTCACATGCTGCATAC |
| 5156 | UK3-41 (N5866) breakpoint 3 FP | AAGGCGTAATGGACACGAGG |
| 5472 | UK3-41 (N5866) breakpoint 3 RP | GCATCGTATTTGCACCGTCC |
| 5476 | ALS159 (N5101) breakpoint 1 FP | tccgcagccgaagttacaat |
| 5477 | ALS159 (N5101) breakpoint 1 RP | acgtcactctctgcccctat |
| 3902 | PtrpC-nat1 FP | CAACTGATATTGAAGGAGCA |
| 1369 | nat-1 RP | GGGCATGCTCATGTAGAGCG |
| 3406 | TERT KO FP1 | AGCGAACTACTACCAACTACG |
| 3407 | TERT KO RP2 | CCCAAAAAATGCTCCTTCAATATCAGTTGGCATGTGAGACACTATCACG |
| 3408 | TERT KO FP3 | CGCTCTACATGAGCATGCCCTGCCCCTGAAAGTCTGGTACTGGGATTGG |
| 3409 | TERT KO RP4 | CTTTCACGACTACCTTCCAAG |
| 6271 | 6271_NCU02840_qPCR_F | CCCTCTCAGACGAGGATATTCA |
| 6272 | 6272_NCU02840_qPCR_R | GCTCTGCTGCTTCTCCTTTAT |
| 6277 | 6277_NCU04720_qPCR_F | TCAGGTTGGAGGAGAGGTATAA |
| 6278 | 6278_NCU04720_qPCR_R | CTATGAGGCCGAAATCCTTGT |
| 6281 | 6281_NCU10030_qPCR_F | CTGCGATCGTAACACTGGATTA |
| 6282 | 6282_NCU10030_qPCR_R | CCGTCCGACATGTAATTACTCAG |
| 6283 | 6283_NCU10031_qPCR_F | CCGATGTCCAGAAGCAGTATATTA |
| 6284 | 6284_NCU10031_qPCR_R | CAGAGCAACTGAGTGGATAGTC |
| 3565 | Telomere 1L qPCR FP | AGCGTTCAAATGCCGTGACCTGT |
| 3566 | Telomere 1L qPCR RP | AGTCCAATGGTGCTAACGGCGA |
| 5408 | 5_his-3_FP | AAGCTTGCCATCTCCACCATC |
| 5409 | 5_his-3_RP | GGGCGTGCACGGCTATGG |
| 5410 | 3_his-3_FP | GGGCCAAGCTACCCCGTC |
| 5411 | 3_his-3_RP | GATCCAATGCGGATGGATTCGC |
| 5412 | T3_his-3_FP1 | CCATAGCCGTGCACGCCCATTAACCCTCACTAAAGGGA |
| 5413 | T7_his-3_RP1 | GACGGGGTAGCTTGGCCCTAATACGACTCACTATAGGG |
| 4776 | his-3 qPCR FP | AACAGCTGAGGGAGCCAATG |
| 4777 | his-3 qPCR RP | GTTTCAGGGGTTTCGTTCGC |
