# Supplementary material for: Telomere repeats induce domains of H3K27 methylation in Neurospora
Source: eLife. 2018 Jan 3;7:e31216. doi: 10.7554/eLife.31216 (PMC5752202; doi:10.7554/eLife.31216)
Supplement: Supplementary file 3. — Plasmids used in this study are identified by number and accompanied by a brief description of how they were utilized. [file elife-31216-supp3.docx]

**Supplementary file 3. List of plasmids**

| Plasmid | Description |
| --- | --- |
| 1991 | pBM61 – *his-3* targeting vector |
| 3110 | LG VIL H3K27me2/3 segment #1 cloned into 1991 |
| 3111 | LG VIL H3K27me2/3 segment #2 cloned into 1991 |
| 3112 | LG VIL H3K27me2/3 segment #3 cloned into 1991 |
| 3113 | LG VIL H3K27me2/3 segment #4 cloned into 1991 |
| 3114 | LG VIL H3K27me2/3 segment #5 cloned into 1991 |
| 3115 | LG VIL H3K27me2/3 segment #6 cloned into 1991 |
| 3116 | LG VIL H3K27me2/3 segment #7 cloned into 1991 |
| 3117 | LG VIL H3K27me2/3 segment #8 cloned into 1991 |
| 3172 | (TTAGGG)_76_ cloned into pCR4-TOPO-TA |
| FGSC #10598 | pAL12-Lifeact – source of *trpC::nat-1* |
