# Supplementary material for: Telomere repeats induce domains of H3K27 methylation in Neurospora
Source: eLife. 2018 Jan 3;7:e31216. doi: 10.7554/eLife.31216 (PMC5752202; doi:10.7554/eLife.31216)
Supplement: Supplementary file 4. — A synopsis of the analyses we used to detect translocation breakpoints. Breakpoints were detected by LUMPY (Layer et al., 2014) and confirmed by PCR (for primer sequences, see Supplementary file 2). [file elife-31216-supp4.docx]

**Supplementary File 4. Chromosome rearrangement breakpoint analyses.**

|  | | Detected by sequencing using LUMPY1 | | |  | Determined by genetic mapping2 |
| --- | --- | --- | --- | --- | --- | --- |
| Strain Name | Collection Number | Breakpoint 1 | Breakpoint 2 | Type | Confirmed by PCR | Location Relative to Chromosomal Loci |
| UK3-41 | N5866 | LG V: 3897192-3897194 | LG V: 5776547-5776549 | deletion | yes | Insertional translocation. Segment of LG VR (including al-3  and pyr-6) is inserted into a distal position in LG VIL (inl-chol-2). |
|  |  | LG V: 5776547-5776549 | LG VI: 105930-105932 | interchromosomal |  |  |
| OY350 | N5862 | not detected | not detected | not applicable | not applicable | Quasiterminal translocation. Distal segment of LG VIL (including chol-2 but not nit-6 or het-8) is translocated to LG IR (distal to un-18). |
| ALS159 | N5101 | LG IV: 1479854-1479856 | LG VI: 4144843-4144845 | interchromosomal | partial: confirmed LG IV | Quasiterminal translocation. Segment of LG IVR (including all  IVR markers except psi) is translocated to the tip of LG VIR. |
| OY329 | N5102 | LG VI: 3451742-3451744 | LG III: 1737664-1737666 | interchromosomal | not applicable | Insertional translocation. Segment of LG VIR (including trp-2 and ws-1) is inserted into LG IIIR. |
|  |  | LG VI: 4200441-4200443 | LG III: 1737671-1737673 | interchromosomal |  |  |
| NM149 | N5857 | LG II: 572091-572133 | LG V: 6421603-6421637 | interchromosomal | yes | Quasiterminal translocation. Segment of LG IIL (including ro-3 and distal markers) is translocated to the tip of LG VR. |
|  |  | LG II: 572078-572097 | LG II: 2851306-2851323 | inversion |  |  |
| OY320 | N5863 | LG VI: 3715970-3715972 | LG III: 5274307-5274309 | interchromosomal | yes | Quasiterminal translocation. Distal segment of LG VIR (not including trp-2) is translocated to the tip of LG IIIR. |
| UK2-32 | N5859 | not detected | not detected | not applicable | not applicable | Quasiterminal translocation. Segment of LG IVR (including nit-3 and distal markers) is translocated to LG VL at or near theNOR. |
| AR16 | N5100 | not detected | not detected | not applicable | not applicable | Pericentric inversion. Large segment of LG I (including ser-3 but not un-3, and extending close to LG IR) is inverted. |
| OY337 | N5858 | not detected | not detected | not applicable | not applicable | Quasiterminal translocation. Segment of LG IIR (including arg-12 and distal markers) is translocated to the tip of LG IVR. |
| 1Coordinates of translocation breakpoints (reported as windows due to ambiguity) were identified using LUMPY v0.2.9 (Layer et al. 2014). PCR was performed across candidate breakpoints to verify their authenticity (primers listed in Table S2). 2In cases in which candidate breakpoints were not identified by sequence analysis, approximate breakponts determined by genetic mapping (Perkins 1997) were utilized. | | | | | | |
